# Optimized methods to measure acetoacetate, 3-hydroxybutyrate, glycerol, alanine, pyruvate, lactate and glucose in human blood using a centrifugal analyser with a fluorimetric attachment

**DOI:** 10.1155/S1463924690000281

**Published:** 1990

**Authors:** R. Stappenbeck, A. W. Hodson, A. W. Skillen, L. Agius, K. G. M. M. Alberti

**Affiliations:** 1 Department of Clinical Biochemistry The Medical School Framlington Place Newcastle-upon- Tyne NE2 4HH UK; 2 Framlington Place Newcastle-upon- Tyne NE2 4HH UK

## Abstract

Optimized methods are described for the analysis of glucose, lactate, pyruvate, alanine, glycerol, D-3-hydroxybutyrate and acetoacetate in perchloric acid extracts of human blood using the Cobas Bio centrifugal analyser. Glucose and lactate are measured using the photometric mode and other metabolites using the fluorimetric mode. The intra-assay coefficients of variation ranged from 0.7 to 4.1%, except with very low levels of pyruvate and acetoacetate where the coefficients of variation were 7.1 and 12% respectively. All seven metabolites can be measured in a perchloric acid extract of 20 μl of blood. The methods have been optimized with regard to variation in the perchloric acid content of the samples. These variations arise from the method of sample preparation used to minimize changes occurring in metabolite concentration after venepuncture.


Journal of Automatic Chemistry, Vol. 12, No. 5 (September–October 1990), pp. 213–220

### Optimized methods to measure acetoacetate, 3-hydroxybutyrate, glycerol, alanine, pyruvate, lactate and glucose in human blood using a centrifugal analyser with a fluorimetric attachment

**R. Stappenbeck, A. W. Hodson, A. W. Skillen**
*Department of Clinical Biochemistry, The Medical School, Framlington Place, Newcastle upon Tyne NE2 4HH, UK*

**L. Agius and K. G. M. M. Alberti**
*Framlington Place, Newcastle upon Tyne NE2 4HH, UK*

*Optimized methods are described for the analysis of glucose, lactate, pyruvate, alanine, glycerol, D-3-hydroxybutyrate and acetoacetate in perchloric acid extracts of human blood using the Cobas Bio centrifugal analyser. Glucose and lactate are measured using the photometric mode and other metabolites using the fluorimetric mode. The intra-assay coefficients of variation ranged from 0·7 to 4·1%, except with very low levels of pyruvate and acetoacetate where the coefficients of variation were 7·1 and 12% respectively. All seven metabolites can be measured in a perchloric acid extract of 20 μl of blood. The methods have been optimized with regard to variation in the perchloric acid content of the samples. These variations arise from the method of sample preparation used to minimize changes occurring in metabolite concentration after venepuncture.*

### Introduction

Economical and specific measurement of the blood concentrations of intermediary metabolites such as acetoacetate, 3-hydroxybutyrate, glycerol, alanine and pyruvate, as well as the more frequent measurement of lactate and glucose, is of interest to the clinical chemist. As some of these metabolites are present in low concentrations in human blood their accurate measurement in small samples (50 μl) requires the sensitivity of fluorimetric methods, the required specificity being achieved by using enzymatic reactions. Using both these techniques with a centrifugal fast analyser, economic handling of large numbers of blood samples can be obtained. Methods for this analyser have been described [1] which were adapted from continuous flow methods [2].

For all methods, the blood samples are deproteinized by treatment with perchloric acid (PCA). Perchlorate, however, interferes with the activity of the enzymes used in the methods and as the perchlorate concentration may vary in the samples, this in turn has an effect on the observed concentration of the metabolite [3]. This paper reports on an attempt to overcome the perchlorate interference and optimize the centrifugal fast analyser methods. To do this, it was necessary to examine the effect of pH and perchlorate on the activity of each of the enzymes using spectrophotometric methods. In the light of these findings existing fluorimetric methods were modified and their performance evaluated. The use of spectrophotometric rather than fluorimetric methods for lactate and glucose was also investigated. From these studies optimized automated methods have been provided whereby unneutralized undiluted PCA extracts of blood can be applied directly to the analyser.

Initially, the effect of pH and perchlorate on the spectrophotometric assay each of the enzymes with each substrate was examined. This established that any effect on the enzyme activity was not simply due to changes in fluorescence. The LKB Reaction Rate Analyser was preferred for these experiments due to the ease in which large changes in sample and reagent volumes can be made.

Secondly, the reaction conditions were altered for the fluorimetric metabolite analyses on the Cobas Bio, taking account of the effects of pH and perchlorate on the spectrophotometric methods.

Thirdly, it was demonstrated that photometric methods were preferable to fluorimetric methods with metabolite concentrations in excess of 500 μM. The latter could have been overcome by using dilutions of the PCA extracts but the aim was to establish a protocol whereby all seven metabolites could be analysed automatically on a single untreated PCA extract.

### Materials

Lithium lactate grade L-X, sodium pyruvate Type II, lithium acetoacetate and L-alanine were obtained from Sigma Ltd (Poole, Dorset, UK); NAD, NADH, NADP, ATP, D,L-3-hydroxybutyrate (monosodium salt), lactate dehydrogenase (pig heart – Cat. No. 107069), hexokinase (yeast – Cat. No. 127817), L-alanine dehydrogenase (B subtilis – Cat. No. 102636), glycerokinase (Candida mycoderma – Cat. No. 127795), 3-hydroxybutyrate dehydrogenase (Rhodopseudomonas spheroides – Cat. No. 737054), glycerol-3-phosphate dehydrogenase (rabbit muscle – Cat. No. 127752) and glucose-6-phosphate dehydrogenase (yeast – Cat. No. 127671) from BCL

0142–0453/90 $3.00 © 1990 Taylor & Francis Ltd.

(Lewes, East Sussex, UK); with glycerol (AR), glucose (50 mmol/l, 5 g/l benzoic acid), perchloric acid – 600 g/kg (AR) and all other chemicals from BDH (Poole, UK).

### Sample preparation

About 1·5–2 ml of whole blood is added to a preweighed plastic tube containing 5 ml of 0·77 mol/l perchloric acid previously cooled to 0 °C. After mixing, the tubes were reweighed, centrifuged at 2500 *g* for 5 min and the protein-free supernatant removed. The dilution of the blood is determined by the weight changes measured. Alternatively, 0·5 ml whole blood may be added volumetrically to 2 ml 0·77 mol/l perchloric acid and the weighing omitted. For the metabolites, other than acetoacetate, analysis of the samples is carried out on the same day or the samples are stored at −20 °C. Acetoacetate must be measured within 3 h of collection of blood or the samples stored at −70 °C.

### Enzyme assay using the LKB Reaction Rate Analyser

To examine the possibility that the apparent difference in metabolite concentration in the presence of variable concentrations of perchlorate is due to interference in fluorescence, rather than by a decreased enzyme activity, measurements were made photometrically at 340 nm using a LKB 8600 Reaction Rate Analyser at 37 °C (Pharmacia LKB Biotechnology Division, Central Milton Keynes, Buckinghamshire, UK). To determine interference by perchlorate, various concentrations were added to cuvettes containing coenzyme in buffered substrate. To initiate the reaction the enzyme or enzymes were injected, usually in a volume of 100 µl. In all cases, the total volume in the cuvettes was 1 ml and the experimental conditions the concentrations referred to are the final concentrations in the cuvettes.

*Acetoacetate*

The cuvettes contained 0·1 mol/l sodium phosphate buffer, pH 7·0 with 310 µmol/l NADH, 40 µmol/l lithium acetoacetate and 25–100 mmol/l sodium perchlorate. 3-Hydroxybutyrate dehydrogenase was added by the pump to give 0·1 U/ml.

*Pyruvate*

The cuvettes contained 0·2 mol/l triethanolamine and 5 mmol/l EDTA, brought to pH 7·4 with 1·0 mol/l HCl, 340 µmol/l NADH. 750 µmol/l sodium pyruvate, and 25–100 mmol/l sodium perchlorate. Lactate dehydrogenase was added by the pump to give 8 mU/mol.

*Lactate*

The cuvettes contained 0·35 mol/l glycine, 0·14 mol/l hydrazinium chloride, 3·64 mol/l EDTA, brought to pH 9·6 with 1·0 mol/l NaOH, 7·5 mmol/l NAD, sodium perchlorate, 25–100 mmol/l and 1 mmol/l lithium lactate. Lactate dehydrogenase was added by the pump to give 50 mU/ml.

*3-Hydroxybutyrate*

The cuvettes contained 0·05 mol/l Tris, 0·5 mol/l hydrazine hydrate, 0·65 mmol/EDTA brought to pH 8·5 with 1·0 mol/HCl, 1·8 mmol/l NAD, 4 mmol/l sodium 3-hydroxybutyrate and 25–100 mmol/l sodium perchlorate. 3-Hydroxybutyrate dehydrogenase was added by the pump to give 30 mU/ml.

*Alanine*

The cuvettes contained 0·02 mol/l Tris, 0·5 mol/l hydrazine hydrate, 0·65 mmol/l EDTA, brought to pH 10 with 1 mol/l HCl, 1·8 mmol/l NAD, 10 mol/l L-alanine and 25–100 mmol/l sodium perchlorate. Alanine dehydrogenase was added by the pump to give 10 mU/ml.

*Glycerol*

The cuvettes contained 0.6 mol/l hydrazine hydrate, 0.12 mol/l glycine, 6 mmol/l $MgCl_2$ brought to pH 9·6 with 1·0 mol/l NaOH, 1·5 mmol/l NAD, 1·6 mmol/l ATP, 1 mmol/l glycerol and 25–100 mmol/l sodium perchlorate. Glycerokinase and glycerol-3-phosphate dehydrogenase were added by the pump to give, respectively, 80 mU/ml and 170 mU/ml. Both these enzymes were diluted from stock in 0·04 mol/l triethanolamine buffer pH 7·4 and injected together.

*Glucose*

The cuvettes contained 0·05 mol/l triethanolamine, 1·4 mmol/l $MgCl_2$ adjusted to pH 8·0 with 10 mol/l HCl, 1·65 mmol/l NADP, 2·4 mmol/l ATP, 2 mmol/l glucose and sodium perchlorate 25–100 mmol/l. Hexokinase, and glucose-6-phosphate dehydrogenase were added by the pump to give 140 mU/ml and 70 mU/ml respectively. Both these enzymes were diluted from stock in 0·04 mol/l triethanolamine buffer pH 7·4 and injected together.

The results for each metabolite are shown in table 1 where it can be seen that the glycerol, acetoacetate and glucose assays are most susceptible to perchlorate.

### pH Optima

When the blood is added to the perchloric acid the acid concentration, as well as the perchlorate concentration of the extract, varies from sample to sample. The extent to

*Table 1. Effect of sodium perchlorate: photometric methods.*

| Metabolite | Perchlorate concentration in cuvette, mmol/l | | | | |
|---|---|---|---|---|---|
| | 0 | 25 | 50 | 75 | 100 |
| | % Activity | | | | |
| Glucose | 100 | 84 | 56 | 41 | 25 |
| Lactate | 100 | 94 | 85 | 78 | 71 |
| Pyruvate | 100 | 98 | 95 | 91 | 89 |
| 3-Hydroxybutyrate | 100 | 90 | 81 | 73 | 69 |
| Glycerol | 100 | 72 | 62 | 50 | 36 |
| Alanine | 100 | 93 | 87 | 81 | 68 |
| Acetoacetate | 100 | 82 | 78 | 64 | 50 |

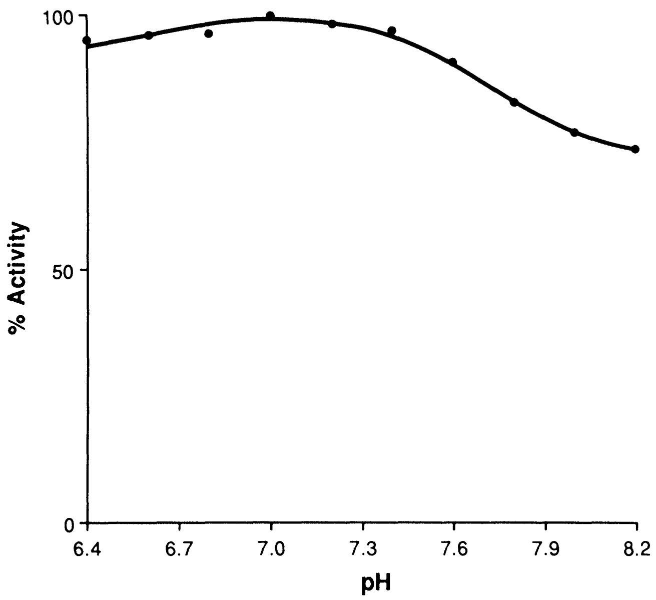

*Figure 1. pH–activity curve for hydroxybutyrate dehydrogenase using acetoacetate as substrate.*

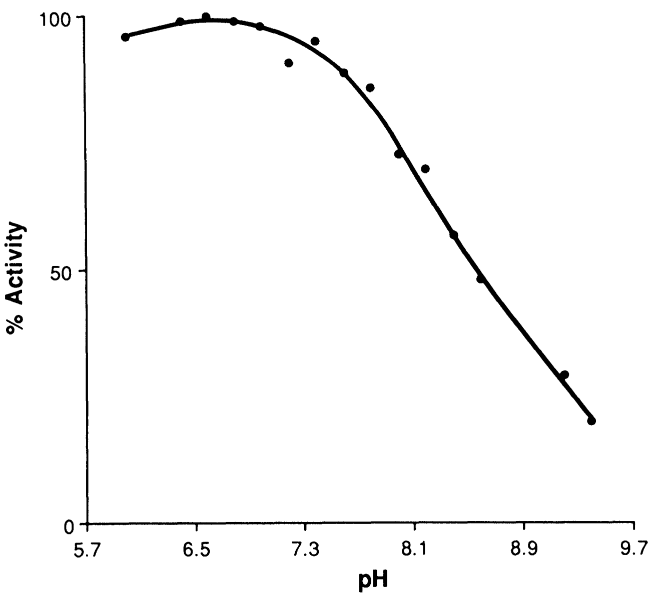

*Figure 2. pH–activity curve for lactate dehydrogenase using pyruvate as substrate.*

which the acidity affects the apparent concentration of a metabolite depends on the activity-pH profile of the enzymes under the experimental conditions used and the buffer capacity. It was necessary therefore to determine the pH profiles for each method to see if the variations in pH found in the PCA extracts would result in relatively large changes in the enzyme activity and hence the apparent metabolite concentration. The pH activity profiles for each assay as determined using the LKB Reaction Rate Analyser are shown in figures 1–7. It is clear that enzymes which show sharp peaks in the activity curves, for example glycerol, will give metabolite assays which are susceptible to variations in the pH of the PCA, particularly with rate reaction methods where the reaction does not go to completion. Clearly, those enzymes which show little change in activity with pH variation will provide metabolite assays much less susceptible to variation in the pH of the extracts.

### Perchloric acid effects in the methods using the centrifugal analyser

The extent to which variations in the perchlorate acid concentrations of the sample combined to alter the apparent concentration of the metabolites were then measured by conventional centrifugal analyser methods. A series of standard solutions of the metabolites were prepared, one set in 0.3 M PCA, one set in 0·4 M PCA, one set in 0·3 M sodium perchlorate, another in 0·3 M hydrochloric acid and a final set in water. The apparent concentrations of the metabolites in these solutions were measured by the methods previously described [1].

Table 2 shows that measurements of 3-hydroxybutyrate, alanine, glycerol and acetoacetate were most affected by perchlorate. An attempt was made to overcome the effect by extending the reaction time, increasing the concentration of the appropriate enzymes, and changing to end-

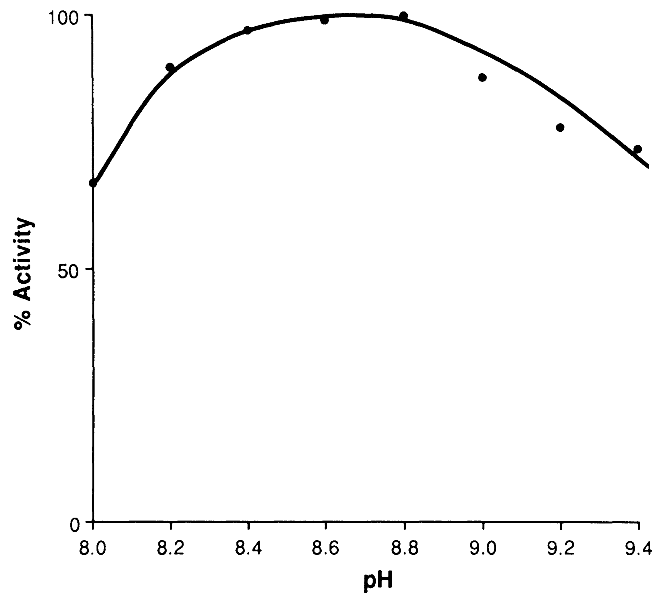

*Figure 3. pH–activity curve for hydroxybutyrate dehydrogenase using hydroxybutyrate as substrate.*

point, rather than initial-rate, methods. For 3-hydroxybutyrate, glycerol and acetoacetate these modifications were largely successful, and the best combination of program parameters are shown in table 3. With the alanine assay, despite a five-fold increase in the incubation time combined with a five-fold increase in the enzyme concentration, complete conversion to pyruvate could not be achieved. The parameters shown in table 3 are therefore those which substantially reduce the effect of variations in PCA concentration and enable alanine concentrations to be measured relatively economically.

As shown in figure 8, fluorescence measurements of NADH and NADPH, at the levels present in the methods

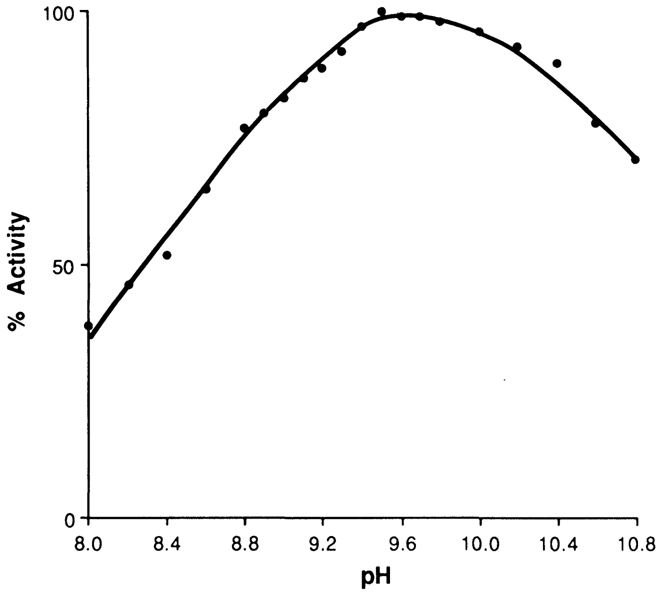

*Figure 4. pH–activity curve for alanine dehydrogenase using alanine as substrate.*

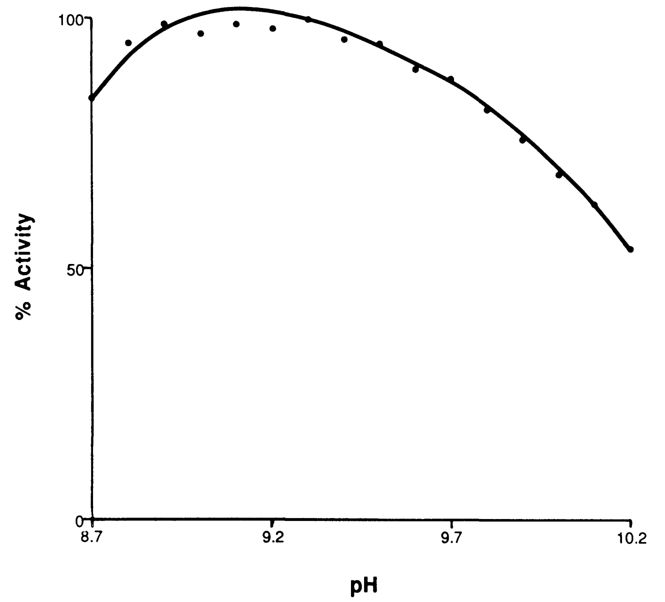

*Figure 5. pH–activity curve for lactate dehydrogenase using lactate as substrate.*

used for measuring lactate and glucose, gave non-linear results with the Cobas Bio. The computer within the instrument has a curve-fitting program, but as this non-linearity could contribute to the overall imprecision in these methods, it was decided to use spectrophotometry rather than fluorimetry for the lactate and glucose analyses.

For optimized direct automated analysis of the metabolites in PCA extracts of blood, the methods are as follows.

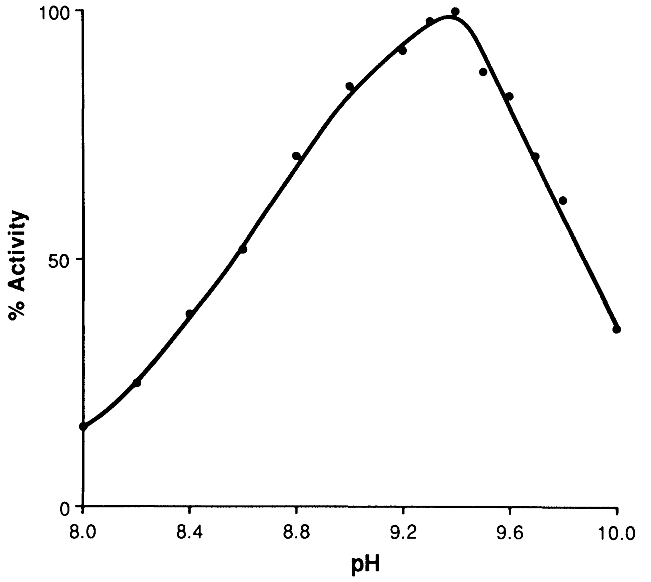

*Figure 6. pH–activity curve for glycerol kinase/glycerol-3-phosphate dehydrogenase using glycerol as substrate.*

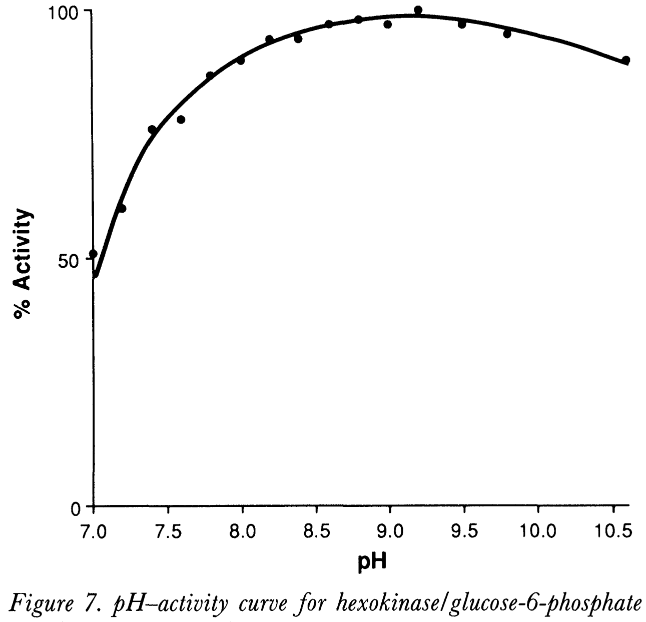

*Figure 7. pH–activity curve for hexokinase/glucose-6-phosphate dehydrogenase using glucose as substrate.*

*Acetoacetate*

Main reagent: 500 μl 0·75 mg/ml NADH in 0·3 mol/l sodium phosphate buffer pH 7·4 is added to 20 ml of the same buffer.

Start reagent: 200 μl 3-hydroxybutyrate dehydrogenase (7·5 U) is added to 400 μl water.

To set the PM voltage use water as sample and a coenzyme reagent containing: 650 μl 0·75 mg/ml NADH in 0·3 mol/l sodium phosphate buffer pH 7·4 added to 20 ml of the same buffer.

*Table 2. Effect of perchloric acid, sodium perchlorate and hydrochloric acid on the apparent concentration of metabolites.*

| Solutions used for preparation of metabolites | Pyruvate 50 µmol/l | Lactate 1000 µmol/l | Glucose 2·50 µmol/l | BOH 100 µmol/l | Glycerol 100 µmol/l | Alanine 250 µmol/l | Acetoacetate µmol/l |
|---|---|---|---|---|---|---|---|
| 0·40 mol/l Perchloric acid | +2 | +1 | +1 | −17 | −14 | +10 | −11 |
| 0·70 mol/l Perchloric acid | +2 | +1 | 0 | −29 | −23 | +10 | −18 |
| 0·40 mol/l Sodium perchlorate | +7 | +3 | 0 | −20 | −15 | −7 | −5 |
| 0·70 mol/l Sodium perchlorate | +9 | +1 | 0 | −30 | −26 | −15 | −16 |
| 0·40 mol/l Hydrochloric acid | −7 | +3 | +1 | −6 | −1 | +15 | −8 |
| 0·70 mol/l Hydrochloric acid | −10 | +2 | +2 | −7 | −2 | +21 | −4 |

The results are expressed as a percentage increase or decrease in the apparent concentration of the metabolite, as compared with that found in aqueous solution of the same concentration.

*Alanine*

Main reagent: 0·04 mol/l TRIS, 1 mol/l hydrazine hydrate, 1·34 mmol/l EDTA (disodium salt) adjusted to pH 10·0 with 10 mol/l hydrochloric acid.

Start reagent: 20 mg NAD and 100 µl alanine dehydrogenase (12 U) per 10 ml 0·1 mol/l phosphate buffer pH 7·4. The working reagent is prepared by adding 0·7 ml of this solution to 8·8 ml of the buffer. This dilution is freshly prepared for each batch of analyses and is warmed to 25 °C before use.

To set the PM voltage use a standard of 350 µmol/l.

*Glucose*

Main reagent: 0·1 mol/l triethanolamine, 2 mmol/l magnesium chloride, adjusted to pH 8·0 with 10 mol/l hydrochloric acid.

Start reagent: 32 mg NADP, 37 mg ATP (disodium salt), 100 µl hexokinase (140 U) and 100 µl glucose-6-phosphate dehydrogenase (70 U) per 10 ml 0·4 mol/l triethanolamine buffer, pH 7·4. This solution is prepared within 1 h of use.

*Glycerol*

Main reagent: 0·2 mol/l glycine, 1 mol/l hydrazine hydrate, 10 mmol/l magnesium chloride brought to pH 9·5 with 10 mol/l sodium hydroxide.

Start reagent: 20 mg NAD, 20 mg ATP (disodium salt), 50 µl glycerokinase (21·3 U) and 100 µl glycerol-3-phosphate dehydrogenase (170 U) per 10 ml 0·4 mol/l triethanolamine buffer, pH 7·4. This mixture is prepared immediately before use and should be at 25 °C when applied to the analyser.

To set the PM voltage use a standard of 140 µmol/l.

*Table 3. Program parameters.*

| | Parameter | ACAC | Pyr | Gluc | Lact | Ala | Glyc | BOH |
|---|---|---|---|---|---|---|---|---|
| | Alpha Code | 13(−) | 13(−) | 11(+) | 11(+) | 11(+) | 11(+) | |
| (1) | Units | 4 | 4 | 3 | 4 | 4 | 4 | 4 |
| (2) | Calculation factor | 0 | 0 | 0 | 0 | 0 | 0 | 0 |
| (3) | Standard 1 conc. | 25 | 10 | ·50 | 200 | 50 | 20 | 20 |
| (4) | Standard 2 conc. | 50 | 30 | 1.50 | 600 | 150 | 60 | 60 |
| (5) | Standard 3 conc. | 100 | 60 | 3·00 | 1200 | 300 | 120 | 120 |
| (6) | Limit | 150 | 65 | 3.2 | 1300 | 320 | 130 | 130 |
| (7) | Temperature, °C | 25 | 25 | 25 | 25 | 25 | 25 | 25 |
| (8) | Analysis mode | 6 | 6 | 6 | 6 | 4 | 6 | 6 |
| (9)* | Wavelength, nm | 340·2 | 340·2 | 340 | 340 | 340·2 | 340·2 | 340·2 |
| (10) | Sample volume, µl | 20 | 10 | 5 | 5 | 10 | 5 | 5 |
| (11) | Diluent volume, µl | 20 | 10 | 25 | 25 | 20 | 25 | 25 |
| (12) | Reagent volume, µl | 160 | 200 | 200 | 200 | 270 | 170 | 170 |
| (13) | Incubation time, s | 120 | 120 | 120 | 120 | 0 | 120 | 120 |
| (14) | Start volume, µl | 20 | 10 | 20 | 20 | 0 | 20 | 20 |
| (15) | 1st reading time, s | ·5 | ·5 | ·5 | ·5 | ·5 | ·5 | ·5 |
| (16) | Time interval, s | 60 | 60 | 60 | 60 | 60 | 60 | 60 |
| (17) | Number of readings | 20 | 5 | 6 | 7 | 20 | 20 | 20 |
| (18) | Blanking mode | 1 | 1 | 1 | 1 | 1 | 1 | 1 |
| (19) | Print-out mode | 1 | 1 | 1 | 1 | 2 | 1 | 1 |
| PMV** | | 380 | 508 | – | – | 532 | 660 | 520 |

All concentrations are in µmol/l except glucose which is in mmol/l.
* The setting 340·2 is used in fluorescence modes to denote excitation wavelength 340 nm with emission filter position 2 (470 nm). The lactate and glucose methods are photometric hence no filter position is required.
** PM voltages are for guidance only and may vary depending on the state of the instrument.

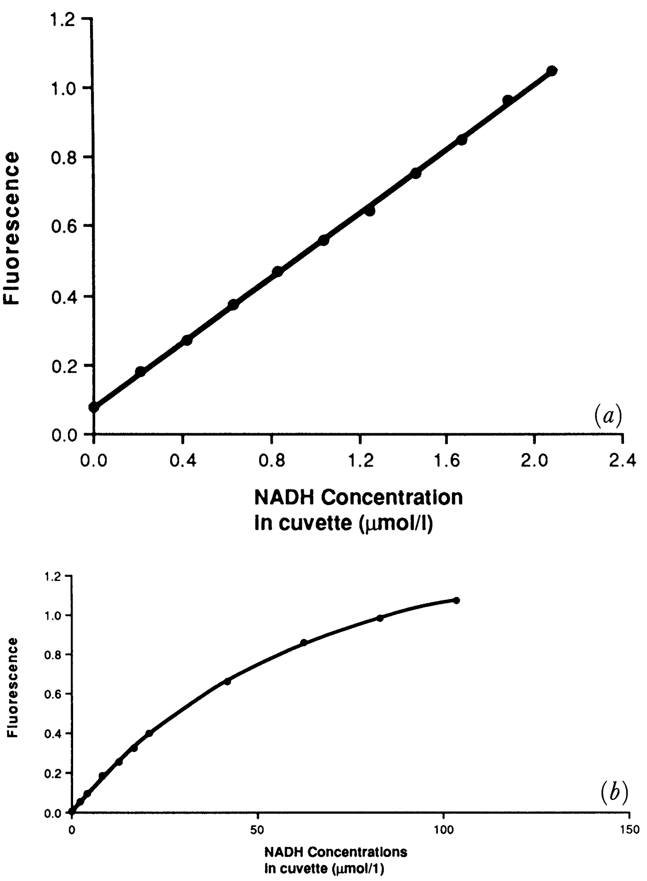

*Figure 8. Relationship between fluorescence intensity and NADH concentration.*
*(a) 0–2.4 µmol/l NADH.*
*(b) 0–100 µmol/l NADH.*

*Pyruvate*

Main reagent: 32 µl mol/l NADH in 0·1 mol/l phosphate buffer, pH 7·4 is added per 10 ml of 0·4 mol/l triethanolamine buffer (0·4 mol/l triethanolamine chloride, 10 mmol/l disodium EDTA adjusted to pH 7·4 with 10 mol/l sodium hydroxide).

Start reagent: 63 µl lactate dehydrogenase (150 U) is added per 10 ml 0·1 mol/l phosphate buffer at pH 7·4. Both coenzyme reagent and start reagent are prepared immediately before use.

To set the PM voltage, use water as sample and a NADH solution 20% more concentrated than used in the assay proper, i.e. 38 µl g/l NADH per 10 ml buffer.

*Lactate*

Main reagent: 0·5 mol/l glycine, 0·2 mol/l hydrazinium chloride, 5·4 mmol/l EDTA (disodium salt) adjusted to pH 9·6 with 10 mol/l sodium hydroxide.

Start reagent: 100 mg NAD and 1 ml lactate dehydrogenase (300 U) per 10 ml 0·1 mol/l phosphate buffer at pH 7·4. This is prepared immediately before use.

*Table 4. Intra-assay imprecision of analyses with optimized methods.*

| | Mean µmol/l | CV | *N* |
|---|---|---|---|
| Alanine | 45·2 | 2·3 | 24 |
| | 203 | 2·0 | 24 |
| 3-Hydroxybutyrate | 20·1 | 4·1 | 21 |
| | 99·3 | 2·6 | 25 |
| Glycerol | 19·9 | 4·4 | 24 |
| | 92·7 | 1·9 | 24 |
| Glucose | 700 | 1·2 | 24 |
| | 2270 | 1·3 | 24 |
| Lactate | 250 | 1·3 | 24 |
| | 1106 | 0·7 | 24 |
| Pyruvate | 7·3 | 7·3 | 25 |
| | 31·7 | 2·5 | 25 |
| Acetoacetate | 16 | 12·0 | 25 |
| | 78 | 2·5 | 25 |

*3-Hydroxybutyrate*

Main reagent: 0·2 mol/l TRIS, 0·1 mol/l hydrazine hydrate*, 2·7 mmol/l EDTA (disodium salt) adjusted to pH 8·9 with 10 mol/l hydrochloric acid.

Start reagent: 20 mg NAD and 1·4 ml 3-hydroxybutyrate dehydrogenase (7·5 U) per 10 ml 0·1 mol/l phosphate buffer, pH 7·4. This is prepared immediately before use.

To set the PM voltage use a standard of 140 µmol/l.

For the assays for alanine, glycerol and 3-hydroxybutyrate, the relatively high blank fluorescence makes it advisable to set the PM voltage using standard solutions of concentrations up to 20% higher than the respective top standards.

*Standard solutions*

Stock standards: 100 mmol/l lithium lactate, 10 mmol/l sodium pyruvate, 10 mmol/l D,L-3-hydroxybutyrate, 100 mmol/l glycerol, 10 mmol/l acetoacetate, 250 mmol/l and 50 mmol/l glucose alanine are prepared in deionized-distilled water and diluted to the appropriate working level with 0·46 mol/l perchloric acid. All standards are stored in a refrigerator at 4 °C for seven days. Working standards containing all seven metabolites at the concentrations shown in table 3 are prepared on a daily basis.

*Cobas Bio parameters for optimized methods*

It was found preferable to use print-out mode 1 for all analyses apart from alanine. With print-out mode 1, the changes in fluorescence are related to the fluorescence of the reaction mixture prior to addition of the start reagent; with print-out mode 2 the changes in fluorescence are related to the fluorescence of the reaction mixture 0·5 s after addition of the start reagent. If very rapid changes in fluorescence occur, such as with samples of high pyruvate concentration, the change in fluorescence in the first 0·5 s

* Although hydrazine has been used in the optimized method it may be omitted on safety grounds without a significant loss of performance.

*Table 5. Effect of perchloric acid, sodium perchlorate and hydrochloric acid on the apparent concentration of metabolites: optimized methods.*

| Solutions used for preparation of metabolites | Pyruvate 50 μmol/l | Lactate 1000 μmol/l | Glucose 2·50 μmol/l | BOH 100 μmol/l | Glycerol 100 μmol/l | Alanine 250 μmol/l | Acetoacetate 100 μmol/l |
|---|---|---|---|---|---|---|---|
| 0·40 mol/l Perchloric acid | +5 | −4 | +1 | −3 | −1 | +10 | −5 |
| 0·70 mol/l Perchloric acid | +3 | −5 | 0 | −6 | −2 | +10 | −3 |
| 0·40 mol/l Sodium perchlorate | +3 | 0 | 0 | −6 | −2 | −8 | 0 |
| 0·70 mol/l Sodium perchlorate | +3 | 0 | 0 | −7 | −3 | −15 | −4 |
| 0·40 mol/l Hydrochloric acid | +3 | −2 | 0 | −4 | −1 | +15 | −6 |
| 0·70 mol/l Hydrochloric acid | +1 | −2 | +2 | −2 | −2 | +21 | 0 |

The results are expressed as a percentage increase or decrease in the apparent concentration of the metabolite as compared with that found in aqueous solution of the same concentration.

is significant and with print-out mode 2 an error is introduced. This and the other changes in parameters are included in table 3 where the parameters used for the optimized methods are listed.

### Evaluation of optimized methods

The intra-assay imprecision is shown in table 4. For alanine, the parameters of the method are the same as those described previously [1], consequently the data for precision were not reassessed.

The effect of perchlorate, PCA and hydrochloric acid in the sample were examined using the optimized methods and the parameters listed in table 3. The concentrations used were within the range likely to be found in extracts using the standard method of sample collection where a variable quantity of blood is added to 5 ml of 0·77 mol/l perchloric acid. The results are shown in table 5.

### Discussion

To determine the *in vivo* concentrations of metabolites accurately it is necessary to make measurements immediately after venepuncture. This can be done with the biosensor systems now available to measure glucose [4] and lactate [5] in whole blood. More usually, blood samples are sent to a laboratory, which is often sited some distance away, so there can be a delay between venepuncture and analysis, incurring changes in metabolite concentrations. Even when fluoride is added to blood, stabilization is neither instantaneous nor complete, although after the first hour the level can be kept relatively stable at 4 °C for about 24 h, followed by a further slow decrease [6]. Complete stability of glucose and other metabolites is obtained if the blood proteins are precipitated and the enzymes activated with PCA. Storage at −20 °C of metabolites in PCA ensures a long period of stability except for acetoacetate where the lower temperature of −70 °C may be required [2].

The main disadvantages of such preparation of samples are that perchlorate and acidity interfere with the activity of the enzymes used in the subsequent methods. As perchlorate affects both photometric and fluorimetric measurement modes, interference in fluorescence characteristics is not the explanation. One way to overcome the problem of PCA can be to neutralize the samples by titration with potassium carbonate or potassium hydroxide, which precipitates the perchlorate. Precipitation may not be complete and the neutralization procedure is in any case time-consuming and increases the imprecision of the technique. In addition it would require a significant amount of sample preparation prior to automated analysis, whereas the present aim was to reduce sample handling to a minimum.

The differences in the perchlorate ion and acid concentration of the perchloric acid extracts can be minimized by pipetting a measured volume of blood into the PCA. However, such a procedure is time-consuming and leads to a delay in stabilization. In addition, the use of pipettes requires some degree of expertise, which those people most likely to be taking blood may not have. Substituting end-point methods for initial-rate methods overcomes the inaccuracy due to the presence of different amounts of PCA, except for alanine. With alanine the large amount of enzyme and the long incubation time required makes end-point analysis unacceptably expensive and time-consuming. Fortuitously, the effects of acidity counteract the effects of the perchlorate ion, so that in practice the initial rate technique is the method of choice for alanine.

In general, end-point methods are less precise than initial-rate methods. This is because the calculations in the former are based on only two measurement, whereas, in the latter, multiple measurements are made. Initial-rate methods also use less enzyme and are quicker, but these advantages can only be utilized when the PCA concentration is exactly the same in samples and standards. In addition, it is necessary to be very precise about the activity of the enzyme used, and this should be determined at frequent intervals. Wide variations can occur in the activities of different batches of nominally identical enzymes provided by the same supplier.

The concentrations of glucose and lactate in blood are such that photometric methods can be used to measure these two metabolites. This is preferred to the alternative of diluting the sample and using fluorescence.

When large numbers of samples are being handled by several persons, the method chosen to measure an analyte is a compromise of various analytical ideals and objectives. These optimized methods have been developed in response to a first priority of minimizing changes in blood metabolites occurring after venepuncture, and, secondly, to improve accuracy. The former requires that sample preparation can be made by persons who do not necessarily have laboratory training and that blood is added to PCA without delay. All the analyses can be carried out without the need of further dilution. For glucose, the imprecision is within the limit now regarded as necessary for contemporary methods [7]. The other methods give a within-batch precision which makes them very useful. Some are slightly more prolonged and costly than those previously described [1], but they now provide more reliable automated assays.

### References

1. Harrison, J., Hodson, A. W., Skillen, A. W., Stappenbeck, R., Agius, L. and Alberti, K. G. M. M., *Journal of Clinical Chemistry and Clinical Biochemistry*, **26** (1988), 141.
2. Lloyd, B., Burrin, J. M., Smyth, P. and Alberti, K. G. M., *Clinical Chemistry*, **24** (1978), 1724.
3. Stappenbeck, R., Hodson, A. W. and Skillen, A. W., *Clinical Chemistry*, **32** (1986), 1023.
4. Matthews, D. R., Holman, R. R., Brown, E., Steemson, J., Watson, A., Hughes, S. and Scott, D., *Lancet* (1987), 778.
5. Clark, L. C., Hoyes, L. K., Grooms, T. A. and Moore, M. S., *Critical Care Medicine* (1984), **12**, 461.
6. Chan, A. Y. W., Swaminathan, R. and Cockram, C. S., *Clinical Chemistry*, **35** (1989), 315.
7. Peake, M. J., Pejakovic, M. and White, G. H., *Clinical Chemistry*, **35** (1988), 404.

